# Analysis of the Promoters Involved in Enterocin AS-48 Expression

**DOI:** 10.1371/journal.pone.0090603

**Published:** 2014-03-04

**Authors:** Rubén Cebrián, Sonia Rodríguez-Ruano, Manuel Martínez-Bueno, Eva Valdivia, Mercedes Maqueda, Manuel Montalbán-López

**Affiliations:** Departamento de Microbiología, Facultad de Ciencias, Universidad de Granada, Granada, Spain; University of Kansas Medical Center, United States of America

## Abstract

The enterocin AS-48 is the best characterized antibacterial circular protein in prokaryotes. It is a hydrophobic and cationic bacteriocin, which is ribosomally synthesized by enterococcal cells and post-translationally cyclized by a head-to-tail peptide bond. The production of and immunity towards AS-48 depend upon the coordinated expression of ten genes organized in two operons, *as-48ABC* (where genes encoding enzymes with processing, secretion, and immunity functions are adjacent to the structural *as-48A* gene) and *as-48C_1_DD_1_EFGH*. The current study describes the identification of the promoters involved in AS-48 expression. Seven putative promoters have been here amplified, and separately inserted into the promoter-probe vector pTLR1, to create transcriptional fusions with the *mCherry* gene used as a reporter. The activity of these promoter regions was assessed measuring the expression of the fluorescent mCherry protein using the constitutive pneumococcal promoter P_X_ as a reference. Our results revealed that only three promoters P_A_, P_2(2)_ and P_D1_ were recognized in *Enterococcus faecalis*, *Lactococcus lactis* and *Escherichia coli*, in the conditions tested. The maximal fluorescence was obtained with P_X_ in all the strains, followed by the P_2(2)_ promoter, which level of fluorescence was 2-fold compared to P_A_ and 4-fold compared to P_D1_. Analysis of putative factors influencing the promoter activity in single and double transformants in *E. faecalis* JH2-2 demonstrated that, in general, a better expression was achieved in presence of pAM401-81. In addition, the P_2(2)_ promoter could be regulated in a negative fashion by genes existing in the native pMB-2 plasmid other than those of the *as-48* cluster, while the pH seems to affect differently the *as-48* promoter expression.

## Introduction

AS-48 is a 70-residue alpha-helical circular cationic bacteriocin ribosomally produced by diverse *Enterococcus* strains, with antimicrobial activity against food-borne pathogenic and food-spoilage bacteria. These characteristics, together with its stability and solubility over wide pH and temperature ranges, confer a clear potential to be used as food biopreservative (reviewed by [1]). Besides this, AS-48 could have veterinary and clinical applications [2] currently under investigation, underscoring its potential as an antimicrobial agent in some disease treatment. For all these reasons, the AS-48 producer strains are of great industrial and pharmaceutical interest and genetic engineering to improve the production of the enterocin AS-48 may be desirable. The conclusive identification of the promoters involved in AS-48 expression and a better understanding of the regulation of the gene expression would facilitate the desired manipulations. Actually, there is extensive and detailed information on the genetic determinants and physicochemical characteristics of AS-48 (reviewed by [3]). The gene cluster involved in AS-48 expression was separately described by Martínez-Bueno *et al.* [4] and Díaz *et al.* [5] in the conjugative, pheromone response plasmid pMB-2 (68 kb), and by Tomita *et al.* [6], who described the identical bacteriocin (namely bac21) located in the pPD1 plasmid (59 kb), both in *Enterococcus faecalis* strains. An additional variant, AS-48RJ produced by *E. faecium* was found to be encoded in the chromosome [7]. More recently, a new AS-48 producer strain, *E. faecalis* UGRA10 carrying a 70-kb plasmid, has been isolated from a Spanish sheep's cheese [8]. Remarkably, *E. faecalis* UGRA10 shows characteristics of a probiotic strain with biotechnological potential to be developed as protective agent in food preservation.

**Figure 1:**
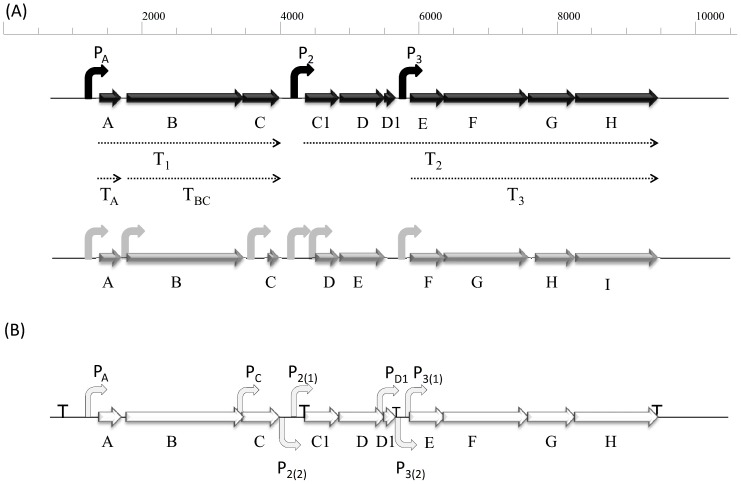
(A) Schematic representation of the *as-48* (black) and *bac21* (grey) gene clusters. Solid black arrows represent the proposed promoter regions and dotted arrows indicate the mRNAs detected by Fernandez *et al.* [10], Díaz *et al.* [5] and Martínez-Bueno *et al.* [9]. Solid grey arrows represent the promoter regions proposed by Tomita *et al.* [6]. (B) Promoters identified *in silico* (dashed arrows) and their location according to AS-48 nomenclature (Genebank KJ146793 and Y12234.1): P_A_ (nt 1105-nt 1396), Pc (nt 2129-nt 2477), P_2(2)_ (nt 2788-nt 3163), P_2(1)_ (nt 2788-nt 3010), P_D1_ (nt 3721-nt 4160), P_3(1)_ (nt 4353-nt 4544) and P_3(2)_ (nt 4188-nt 4544). Predicted terminators according to BPROM [32] in *as-48* gene cluster are pointed with a **T**.

According to Martínez-Bueno *et al.* [4] and Díaz *et al.* [5] the full expression of the *as-48* cluster depends on the co-ordinated expression of ten genes (*as-48A, B, C, C_1_*, *D, D_1_, E, F, G* and *H*) (GenBank accession number KJ146793, Y12234.1 and AJ438950.1), although only nine (*bacA, B, C, D, E, F, G, H*, and *I*) were identified in the *bac* cluster [6] (Genbank D85752.1) (Figure 1). However, in the physical and genetic map published by each group there are some differences (Figure 1A). The main discrepancy is that in the *bac* cluster a protein homologous to As-48D_1_, the proposed immunity determinant against AS-48, was not considered. However, the mutants that were described in that work show that the deletion of the region where the immunity protein As-48D_1_ is encoded, clearly makes a difference in the phenotype in terms of resistance against AS-48 [6]. This is consistent with the existence of a small ORF encoding an immunity determinant as was shown later [9]. There are some other variations, in addition to the nomenclature used, related to the predicted initiation codons for *bacC* and *bacD* genes (homologous to *as-48C* and *as-48C_1_*, respectively), and also on a putative promoter proposed for the *bacC* gene (Figure 1A).

Transcriptional analysis of the *as-48* cluster revealed the existence of two polycistronic mRNAs, T_1_ (3.5 kb) and T_2_ (6.4 kb), corresponding to the expression of the two operons *as-48ABC* and *as-48C_1_DD_1_EFGH*, respectively (Figure 1A). A post-transcriptional regulation mechanism was elucidated for T_1_ that undergoes endonucleolytic processing into two smaller fragments with different half-life in order to ensure the optimal stoichiometry of each gene product [10]. Furthermore a second and shorter mRNA (T_3_, 5.4 kb), possibly transcribed from an internal promoter, encodes at least the last four genes (*as-48EFGH*) [5].

All the commented features are in agreement with the general trend in bacteria, where numerous genes are organized in operons transcribed from the same promoter into a single polycistronic mRNA molecule, although many genes could also be transcribed from internal promoters located at intergenic regions or within adjacent genes [11]. Nevertheless, it has been suggested that T_1_ and T_2_ are constitutively expressed, while transcription from putative internal promoters might be regulated [5]. In other circular bacteriocins like uberolysin, circularin A and butyrivibriocin AR10 there are regulatory elements encoded in the same gene cluster [12], whereas the production of subtilosin A is controlled by external regulators in response to environmental factors [13]–[15].

Our group has provided valuable information regarding the impact of the amino acids in the propeptide sequence that are involved in the head-to-tail peptide bond formation [16] and the impact of circularization in the activity and structure of AS-48 [17], [18]. In this moment, we are interested in unravelling the interactions between the proteins encoded in the *as-48* gene cluster and in elucidating the regulation of the gene expression. Thus, an accurate identification of the promoters is crucial. Such information could also help to explain the failure in the heterologous expression of AS-48 in other lactic acid bacteria, especially in *Lactococcus lactis* [19], a GRAS (*generally recognized as safe*) bacterium of great biotechnological interest. Additionally, the identification of promoters, particularly those strong and inducible, provides a potent biotechnological tool for research and industry [20]–[23]. However, the identification of promoter regions is problematic when dealing with bacterial genomes that have a high A+T content such as *E. faecalis* (ca. 60% A+T). In these genomes, stretches resembling −10 elements (5′-TATAAT-3′) are frequent and, therefore, the definitive identification of promoters from sequence information remains more difficult [11]. For these reasons, we have investigated the activity of the several putative *as-48* promoter regions identified *in silico* measuring their different expression level in diverse strains. For this, we carried out transcriptional fusions of each putative promoter fragment to drive the expression of a synthetic *mCherry* gene codon-optimized for *Enterococcus* into the pTLR1 vector [24]. We isolated seven putative promoter fragments from the *as-48* cluster according to two software analyses and cloned each fragment to drive the expression of the *mCherry* reporter gene in three bacterial strains, *E. faecalis*, *L. lactis* and *E. coli*, using the previously characterized promoter P_X_ of *Streptococcus pneumoniae* for comparison [24].

## Materials and Methods

### Bacterial strains, vectors and culture conditions

Bacterial strains and vectors used in this work are listed in Table 1. *Escherichia coli* was grown at 37°C with shaking in Luria broth (LB; Scharlau, Barcelona, Spain) and selected with erythromycin (Em 250 µg/ml, Sigma-Aldrich, Madrid, Spain) for cells harboring the pTLR1-derivatives. *E. faecalis* and *L. lactis* were routinely grown in brain heart infusion (BHI; Scharlau) at 37°C and M17 (Scharlau) plus glucose (0.5%), GM17, at 30°C, respectively. Erythromycin at 10 µg/ml and/or chloramphenicol at 20 µg/ml (Sigma-Aldrich, Madrid, Spain) were added to the culture medium for cells harboring pTLR1 derivative plasmids or pAM401-81 plasmid, respectively.

**Table 1:** Strains and plasmids used in this study. Cm^R^ chloramphenicol resistant, Em^R^ erythromycin resistant.

Bacteria	Characteristics	Source
*Escherichia coli* TOP10	F- *mcrA Δ(mrr-hsdRMS-mcrBC) φ80lacZΔM15 ΔlacX74 nupG recA1 araD139 Δ (ara-leu)7697 galE15 galK16 rpsL*(Str^R^) endA1 λ^−^	Invitrogen
*Enterococcus faecalis* JH2-2	Plasmid free, Rif^r^, Fus^r^, AS-48^s^	[42]
*Enterococcus faecalis* JH2-2 (pMB-2)	JH2-2 transconjugant with pMB-2 plasmid	[5]
*Lactococcus lactis* LM2301	Plasmid free, host strain for cloning	[43]
**Plasmid**	**Characteristics**	**Source**
pAM401-81	Cm^R^, *as-48* gene cluster cloned in pAM401 (25 kb)	[5]
pTLR1d	Obtained from pTLR-1 vector after digestion with *Bgl*II and *Bam*HI (lacking the polylinker but containing the *mCherry* gene)	This work
pTLR1	pTLR derivative containing the promoter P_X_; Em^R^ (8.3 kb)	[24]
pTLR1-P_A_	Em^R^, P_A_ promoter cloned in substitution of Px, controlling *mCherry* expression (8.0 kb)	This work
pTLR1-P_C_	Em^R^, P_C_ promoter cloned in substitution of Px, controlling *mCherry* expression (8.1 kb)	This work
pTLR1-P_2(1)_	Em^R^, P_2(1)_ promoter cloned in substitution of Px, controlling *mCherry* expression (8.0 kb)	This work
pTLR1-P_2(2)_	Em^R^, P_2(2)_ promoter cloned in substitution of Px, controlling *mCherry* expression (8.2 kb)	This work
pTLR1-P_D1_	Em^R^, P_D1_ promoter cloned in substitution of Px, controlling *mCherry* expression (8.2 kb)	This work
pTLR1-P_3(1)_	Em^R^, P_3(1)_ promoter cloned in substitution of Px, controlling *mCherry* expression (8.0 kb)	This work
pTLR1-P_3(2)_	Em^R^, P_3(2)_ promoter cloned in substitution of Px, controlling *mCherry* expression (8.15 kb)	This work

For fluorescence detection, several culture media were assayed: the chemically defined media CDM-PC [25] and CDM-BP [26], the semi-defined complex medium supplemented with 0.8% glucose (CM-G) [27] and the complex media GM17 and LB.

### General DNA manipulation and transformation

The plasmid-free strain *E. coli* TOP10 was used in cloning experiments. The preparation of chemiocompetent cells to be transformed with plasmid DNA and ligation products was done by the calcium chloride protocol as described by Seidman *et al*. [28]. Electroporation of *L. lactis* and *E. faecalis* was performed according to the methods described by Holo and Nes [29] and Friesenegger *et al.* [30], respectively. Plasmid DNA was isolated from *E. coli* using the Plasmid Mini I kit from Omega bio-tek (VWR International, USA). PCR products were purified with the AccuPrep PCR Purification Kit (Bioneer, Daejeon, Korea) and sequenced. Restriction enzymes were obtained from Thermo Scientific (Madrid, Spain), ligase from Invitrogen (Life Technologies, Madrid, Spain), TaqDNA polymerase from MBL (MBL International Corporation, Woburn, USA); and used as recommended by the suppliers. DNA was sequenced using an ABI PRISM Dye Terminator Cycle Sequencing Ready Reaction (Perkin Elmer, Applied Biosystems, USA).

### *In silico* analysis

Putative promoter regions from *as-48* or *bac* regions (GenBank KJ146793 and Y12234.1, and D85752.1, respectively [9], [6]) were analysed with the bioinformatic programs Promoter Prediction by Neural Network (NNPP) [31] (http://s.fruitfly.org/seq_tools/promoter.html) and BPROM (Softberry Inc., Mount Kisco, NY, USA; http://linux1.softberry.com) [32].

### Construction of the pTLR1-derivative plasmids with the *mCherry* reporter gene

pTLR1 (KitMygen, Madrid, Spain) is a vector for promoter analysis that contains the strong promoter P_X_ from *S. pneumoniae* upstream of *mCherry* [24]. The plasmid pAM401-81 was used as a template for PCR amplification of the different predicted promoters [5], [9]. All primers used in PCRs (listed in Table 2) were synthesized by Biomedal S.L. (Sevilla, Spain) and were based on the published DNA sequence of the *as-48* locus of *E. faecalis* (Genebank KJ146793, Y12234.1 and AJ438950.1). The PCR conditions were the same for all the amplifications performed: 96°C 2′, 30× (96°C 30”, 50°C 30”, 72°C 30”), 72°C 2′. The amplified DNA fragments containing the presumed promoter regions were cut with *Bgl*II and *Bam*HI and ligated into pTLR1 previously digested with the same enzymes, obtaining the pTLR1-derivative constructions shown in Table 1. The ligation mix was transformed into *E. coli* TOP10. The desired orientation of the fragments was determined by colony PCR using the forward primer of each promoter and the pTLR-rev primer, which anneals in the vector backbone (Table 2). The verified plasmid isolated from *E. coli* was used to transform *L. lactis* LM2301 and *E. faecalis* JH2-2 or JH2-2 harboring either pMB-2 or pAM401-81 plasmids. pTLR1d lacking the polylinker but containing the *mCherry* gene without promoter was here obtained from pTLR1 vector after digestion with *Bgl*II and *Bam*HI enzymes and religation.

**Table 2:** Oligonucleotides used in this study.

Target	Primer	Sequence* 5′-3′	Product size (bp)
P_A_	ForP_A_	ACAA*AGATCT* **GCCATGATTGATGAAAAAAA**	256
	RevP_A_	TTTT*GGATCC* **TGCATTTCATTGCTATTATAC**	
Pc	ForP_C_-P_2(1)_	ACGT*AGATCT* **GTACATGCGATTAGATACCATTAATTTTG**	347
	RevPc	CATC*GGATCC* **TAAAAGTTCTATAAAAAAATGTGGAAG**	
P_2(1)_	RevP_2(1)_	TTTT*GGATCC* **CTTTCTTAAGAACTTATATGG**	263
	ForP_2_	TTAC*AGATCT* **TGCTGAGTTAAAGGTATACTC**	
P_2(2)_	ForP_2_	TTAC*AGATCT* **TGCTGAGTTAAAGGTATACTC**	396
	RevP_2(2)_	TACG*GGATCC* **TAATTTAGGAAAAAAACTCAAGTTTTTTTC**	
P_D1_	RevP_D1_	TAGT*GGATCC* **TTCAGTTTGTCAAGATTAATTA**	439
	ForP_D1_	ACGT*AGATCT* **GAATATGACGGCACATTGTATACAG**	
P_3(1)_	ForP_3(1)_	AATT*AGATCT* **AAAATAAGAAGCTGTACAATAG**	191
	RevP_3_	TTTT*GGATCC* **CTTTCTTGTCATAATTAAAG**	
P_3(2)_	RevP_3_	TTTT*GGATCC* **CTTTCTTGTCATAATTAAAG**	356
	ForP_3(2_)	CCGA*AGATCT* **GAATTGATTACATTATTATTATAGTCTCAC**	
pTLR plasmid	pTLR-rev	GTTGAAACTCGTGCGATCCCCCGGG	

*Restriction enzyme sites are depicted in italics.

### Detection and quantification of fluorescence emission in microtiter plates

Each assay was repeated in triplicate in a 96-well optical flat-bottom microplate (Nuclon Delta Surface, Thermo Scientific, Roskilde, Denmark) and monitored with an Infinite 200 Pro microplate spectrophotometer (Tecan Group Ltd., Mannedorf, Switzerland). Briefly, each transformant was separately inoculated into three wells with a final volume of 100 µl/well of the CM-G medium with the appropriate antibiotics at an OD_600 nm_ 0.05 and then grown for 18 h at 30°C for *Lactococcus* or 37°C for *E. coli* and *Enterococcus*. During cultivation the spectrophotometer simultaneously provided quantitatively online data every 10 minutes of cell density (OD_600_) and *in vivo* mCherry fluorescence measured at an excitation wavelength of 590 nm and an emission wavelength of 620 nm. *E. coli* cultures were grown with continuous shaking and stopped 1 min before measuring the OD and fluorescence. In the case of *E. faecalis* and *L. lactis* shaking was applied only for 10 second before taking the measurements of OD and fluorescence.

In order to determine the influence of the pH, the medium was adjusted at different pH values (6, 6.5, 7.0, 7.5 and 8.0) using 0.1 M phosphate buffer according to Gomori [33]. All the experiments were performed in triplicate. The background fluorescence of the control strains (harboring the pTLR1d promoterless plasmid) was subtracted for each time point during the growth. In addition the values of fluorescence shown were normalized by the OD to avoid differences in the growth that may lead to erroneous conclusions.

### Statistical analysis

The statistical analysis of the data was performed using the IBM SPSS statistics 20 (IBM, Spain). Data relating to microbiological density and fluorescence under different conditions were subjected to ANOVA. Tukey was used as a post-hoc test to determine significant differences between promoters and a 0.05 signification level (*p* value) was considered. The average data from duplicate trials ± standard deviation was determined.

### Fluorescence microscopy

Cells were grown overnight in 1 ml of the appropriate medium and harvested by centrifugation. After 3 washes in sterile PBS (Sigma-Aldrich, Madrid, Spain), 5 µl of cells were placed on slides and observed in an Olimpus BX51 microscopy (model BX51TF with a power supply unit Olimpus U-RFL-T SN 1101008) using a TRITC filter (excitation 590 nm and emission 620 nm). The images were taken at 250 ms of excitation.

### Activity tests

The antibacterial activity of diverse AS-48-producing *E. faecalis* strains was performed as described by Fernández *et al.* [18].

## Results and Discussion

### Bioinformatic location of promoter regions in the *as-48* gene cluster

In previous works (e.g. [4]–[6]) several promoter regions triggering the *as-48/bac21* gene cluster expression have been proposed (Figure 1). To unambiguously define the promoter regions involved in AS-48 expression, putative −10 and −35 hexamers were located by their resemblance to the previously defined *Enterococcus* consensus sequences, using the Promoter Prediction by Neural Network (NNPP) [31] and the BPROM programs (Softberry Inc., Mount Kisco, NY, USA; http://linux1.softberry.com) [32]. Preference was given to motifs that matched to the consensus sequence at the most conserved positions of the hexamers and gave rise to a −35/−10 with a 17±1 nt spacer according to both programs. Therefore, a set of seven putative promoters was predicted (Table 3 and Figure 1B). Detection of other promoter regions binding different σ factors in which the −35 sequences are not required or extracellular function σ factors which do not bind a standard −10 sequences could not be achieved using this software. In general, the −10 sequences are better conserved in all the proposed regions while the −35 sequences were substantially less conserved (Table 3). A characteristic TG motif of Gram-positive bacteria promoters, often found 1 bp upstream of the −10 sequence (the −16 region) [34], was found in 4 of the 7 promoters studied (Table 3). Also an AT-rich region located upstream from the −10 and −35 hexamers was identified. Such an AT-rich region may activate the promoter by DNA bending [35] or form an UP element that stimulates transcription through a direct interaction with the C-terminal domain of the RNA polymerase alpha subunit [36]. In general, the distance between the −10 and −35 hexamers ranged from 11 to 18 bp (Table 3).

**Table 3:** Nucleotide sequences of the predicted promoters from the *as-48* gene cluster.

Promoter	−35	Spacing and TG motif	−10	RBS and +1
***consensus***	**TTGACA**		**TATAAT**	**AGGAGG**
**P_A_**	**TTG**cat	CAAAATAAACTACA**TG**GG	**TATAAT**	(30 nt)AGGAGGA(5 nt)ATG
**P_C_**	**TT**tt**C**t	GGGAG**TG**TTAGTAGG	**TATAAT**	(246 nt) AGGA(14 nt)ATG
**P_2(1)_**	**TTG**gg**A**	TAGGCAACTATATTC	**TA**a**AAT**	(56nt)AGGAAG(6 nt)TTG
**P_2(2)_**	**TT**c**AC**t	ATTTTTTT**TG**TTTTCAA	**T**t**TAAT**	(51 nt)AGGGA(20 nt)ATG
**P_D1_**	**TTG**tag	AATATT**TG**TCAAA	**TATAAT**	(17 nt)AGGGA (16 nt)ATG
**P_3(1)_**	**T**a**GAC**t	AATCAGCAAAGGGAGTAT	a**ATAAT**	(108 nt)AGGA(9 nt)ATG
**P_3(2)_**	**TT**tttt	TTCTTCCCCAT	**TA**a**AAT**	(220 nt)AGGA(9 nt)ATG

The putative ribosome binding sites (RBS), TG motif and the distance to −10 boxes and +1 position are shown.

As a result, we have selected the following promoter regions putatively involved in the expression of the AS-48 character (Figure 1 and Table S1):

i) The P_A_ promoter: it is a region with canonical −10 and −35 regions, separated by a correct spacing that could allow for the binding of the vegetative σ factor of the bacterial RNA polymerase without the need for an activator [10].

ii) The P_C_ promoter: Fernández *et al.* [10] reported that *as-48BC* genes overlapped and they had a coupled transcription from the P_A_ promoter in absence of a specific promoter for *as-48B*. This fact does not invalidate, however, the possibility that *as-48C* could have its own promoter (P_C_) as it had been proposed for its counterpart *bacC* [6]. In such case, *bacC* would encode a shorter protein (57 residues), starting 246 nt downstream from *bacB*, which does not overlap with *bacB* as, in fact, it was proposed for its homologous *as-48C* in the *as-48ABC* operon [9]. To address this point, we have amplified the region located 347 nt upstream the *as-48C* gene. It contains motifs that match the consensus sequence at the most conserved positions of the hexamers and an appropriate spacer in accordance with the two predictive programs used (Table 3).

iii) The P_2_ promoter: according to Díaz *et al.* [5], this promoter drives the expression of the *as-48C_1_DD_1_EFGH* operon. However, there are discrepancies between Martínez-Bueno *et al.* [9] and Tomita *et al.* [6], who place the origin of *as-48C_1_* or *bacD* 400 bp and 500 bp, respectively, from the previous predicted ORF. Additionally, the software used in the predictions also shows two possible promoters in the region. Therefore, we decided to clone the two putative promoters, namely P_2(1)_ and P_2(2)_, to clarify this point (Table 3 and Figure 1B).

iv) The P_D1_ promoter: the possibility of an internal promoter in the *as-48C_1_DD_1_EFGH* operon, from which the gene *as-48D_1_* might be transcribed has been also investigated. This new promoter might be located in a 439 nt fragment upstream from the start codon of *as-48D_1_* containing a −10 and a −35 consensus sequence with 4 out of 6 matches (Table 3). This putative internal promoter, which was not considered in the *bac21* cluster [6], would confer some degree of immunity to the producer strain (reviewed by [3], [12]).

v) The P_3_ promoter: the putative P_3_ promoter firmly postulated by both, Díaz *et al.* [5] and Tomita *et al.* [6], driving the expression of the four overlapped *as-48EFGH* genes should be located in the 204 nt intergenic region identified between the *as-48D_1_* and *as-48E* genes, where a plausible −10 and −35 region separated by a correct spacing was found (promoter P_3(1)_) (Table 3). Additionally, a larger region including part of *as-48D_1_* and a series of conserved sequences separated by 9 nt (named promoter P_3(2)_) has been also cloned according to the prediction.

### Construction of pTLR1-derivatives containing the promoter regions fused to the *mCherry* gene

To map more precisely the promoter regions driving the expression of *as-48ABC, as-48C, as-48C_1_DD_1_EFGH*, *as-48D_1_* and *as-48EFGH* genes, the presumed promoter fragments were amplified and separately inserted into the promoter-probe vector pTLR1, creating several transcriptional fusions with the *mCherry* gene, which is codon optimized for expression in LAB [24]. To amplify such regions, we used the pAM401-81 plasmid as template and specific pairs of primers (Table 2) discarding the putative RBS of each promoter in the amplifications. In this way, the RBS is common in all the constructions and therefore the amount of mCherry produced will correlate more accurately with the strength of each promoter without any effect of the individual RBSs. The advantage that this expression system provides is the easy monitoring of the mCherry expression as autofluorescence emitted after an excitation pulse of light with a wavelength of 590 nm. As positive and negative controls, the strong P_X_ promoter from *S. pneumoniae*, which in the absence of the pneumococcal MalR regulator is constitutively expressed [24] and the pTLR1d vector here constructed were used. The recombinant pTLR1-derivative plasmids were separately constructed and cloned into *E. coli* TOP10, and transferred to the LAB hosts *L. lactis* LM2301 and the well-characterized laboratory strain *E. faecalis* JH2-2.

### Conditions for the evaluation of the promoter regions

The mCherry expression can be detected in different ways [20]. As a first indication, a distinct colour of the colonies on agar plates is observed according to the host used: the strongest colour appeared in *E. coli*, being paler in the LAB hosts indicating roughly the strength of the promoters cloned. This result was confirmed by microscopic analysis during both exponential and stationary phase (data not shown). However the most quantitative results were obtained with *E. faecalis*, *L. lactis* and *E. coli* transformants by measuring the simultaneous cell growth and fluorescence during prolonged cultivations using a microplate reader spectrophotometer. To optimize the cultural conditions, we assayed two chemically defined media (CDM-PC and -BP), a semi-defined complex medium (CM-G) specifically designed for AS-48 production and purification purposes, and two complex media (GM17 and LB). The basal arbitrary units of fluorescence (AU) detected before inoculation for CDM-PC (315.8), CDM-BP (316.3), CM-G (401.0), LB (695.8) and GM17 (2943.0), revealed that although CM-G had higher background fluorescence than the CDMs, this medium allows the growth of the different strains to higher OD. Therefore, we chose CM-G for the fluorescence assays.

### Identification of functional promoters in the *as-48* gene cluster

**Figure 2:**
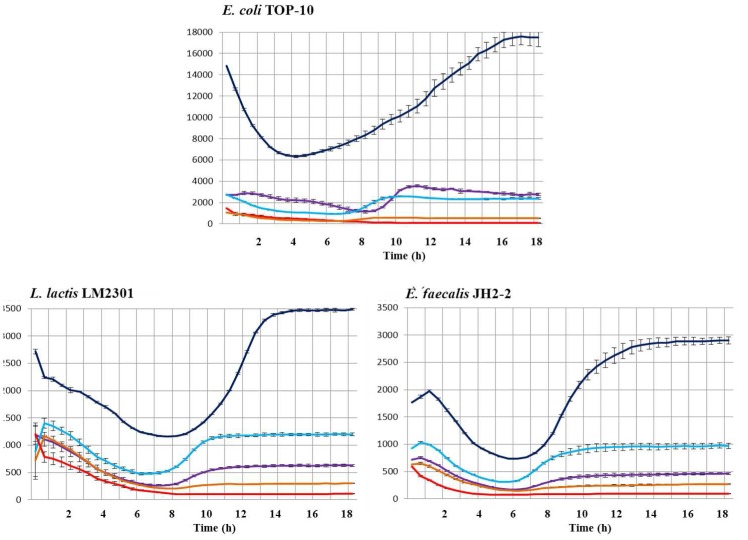
Expression of mCherry normalized by the OD 600-derivatives in *E. coli* TOP10, *L. lactis* LM2301 and *E. faecalis* JH2-2 during prolonged growth in CM-G medium. Fluorescence emission of mCherry was recorded at 620-P_X_ (dark blue), pLTR1-P_A_ (purple), pLTR1-P_2(2)_ (sky blue), pLTR1-P_D1_ (orange) and pTLR1d (red) used as negative control. Standard deviation bars for the different replicates are included.

Taking together our results about the fluorescence emitted in CM-G medium by the transformants with the different promoter regions cloned (Figure 2), we can confirm the existence of P_A_ and the suitability of the P_2(2)_ proposed by Tomita *et al.* [6]. These two promoters are functional and active in *E. coli* and in the two lactic acid bacteria investigated. Apart from this, our results confirm the existence of the internal P_D1_ promoter suggested by Martínez-Bueno *et al.* [9]. The absence of fluorescence observed in the micrographs and during the growth curves, unequivocally confirms that those fragments cloned as P_C_, P_2(1)_, P_3(1)_ and P_3(2)_, did not express the mCherry protein in any of the culture conditions assayed, being for this reason discarded (data not shown). P_A_, P_2(2)_ and P_D1_ contain the typical −16 region, reinforcing the interest of this region for the promoter activity. The absence of P_C_ is in accordance with the expression pattern observed by Fernández *et al.* [10] for the *as-48ABC* operon. In this operon, the P_A_ promoter controls the normal expression of *as-48-ABC* genes rendering the transcript T_1_ that undergoes a post-transcriptional processing, and arises two different transcripts T_A_ and T_BC_ with distinct half-life and stability, ensuring the appropriate stoichiometry of the different gene products (Figure 1A). Additionally, the existence of the promoter P_C_ proposed by [6] would mean that *bacC* is an ORF shorter than *as-48C* and would not match with the typical DUF95 domain found in circular bacteriocin gene clusters.

The absence of mCherry expression driven from P_3_ suggests that if P_3_ does not exist, the second and shorter mRNA (T_3_, 5.4 kb) identified by Díaz *et al.* [5] that encodes the last four genes (*as-48EFGH*), could only be explained by transcription from the internal P_D1_ promoter here identified. This conclusion is in accordance with the loss of transcription observed in JH2-2 (pAM401*_EH_*) transformants where the *as-48A-D_1_* genes were deleted [5]. These results also indicate that most likely there is no monocistronic mRNA encoding for As-48D_1_ as it was suggested by [9] and that the immunity determinants As-48D_1_EFGH are transcribed together since no P_3_ promoter could drive the expression of the T_3_ detected by [5].

**Figure 3:**
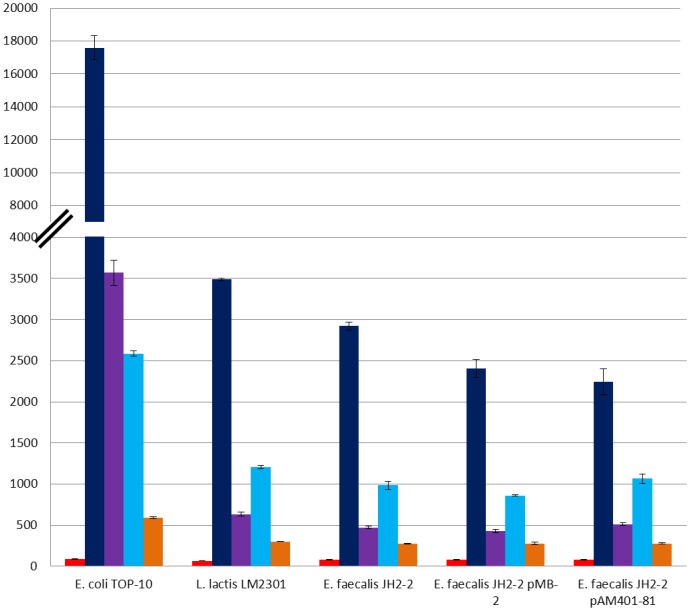
Maximal fluorescence values of the mCherry protein reached during prolonged growth in CM-G medium normalized by the OD 600 nm in single and double transformants bacteria, harbouring the functional derivatives pTLR1-P_X_ (dark blue), pTLR1-P_A_ (purple), pTLR1-P_2_ (sky blue) pTLR1-P_D1_ (orange), and pTLR1d used as negative control (red). Standard deviation bars for the different replicates are included.

As it is shown in Figure 2, the P_X_ promoter from *S. pneumoniae* was the strongest one in *E. coli* as well as in both, *L. lactis* and *E. faecalis*, under these experimental conditions. It is worth noting that among the three functional *as-48* promoters identified in this work, P_2(2)_ directs the highest levels of transcription with maximal fluorescence values ranging from 2585 AU in *E. coli* to 984 AU in *E. faecalis* or 1206 AU in *L. lactis*, being *E. coli* the exception where P_A_ reaches a maximum fluorescence value of ca. 3600 AU (Figure 3). All this is in accordance with the colour of the colonies in solid media and the fluorescence of the cells observed in the fluorescence microscopy (data not shown). As expected, the transcriptional fusion of P_A_ with the *mCherry* gene displayed fluorescence in the LAB species, although surprisingly it was more efficient in lactococcal cells (633 AU *versus* 466 AU). Finally, the P_D1_ promoter shows a basal and maintained expression, of around 25% compared with that of P_2(2)_ in LAB or 20% in case of *E. coli*. This expression level must be enough to ensure its protective functional role in the cells (Figures 2 and 3), together with the expression of the additional determinants As-48EFGH [5], and with As-48C [19], which contains a DUF95 domain recently suggested to be involved in both, production of and immunity, against the circular bacteriocin lactocyclicin Q [37]. In general, the level of fluorescence reached by LAB strains containing pTLR1-P_2(2)_ was 2-fold compared to pTLR1-P_A_ and 4-fold compared to pTLR1-P_D1_. Although the level of expression was lower in LAB strains than in *E. coli*, the ratio between each promoter was maintained. Our cumulative results indicate that the mCherry fluorescence increased in parallel with OD600 during the exponential phase of growth reaching the maximal values during the transition to stationary phase.

### Induction of mCherry expression

**Figure 4:**
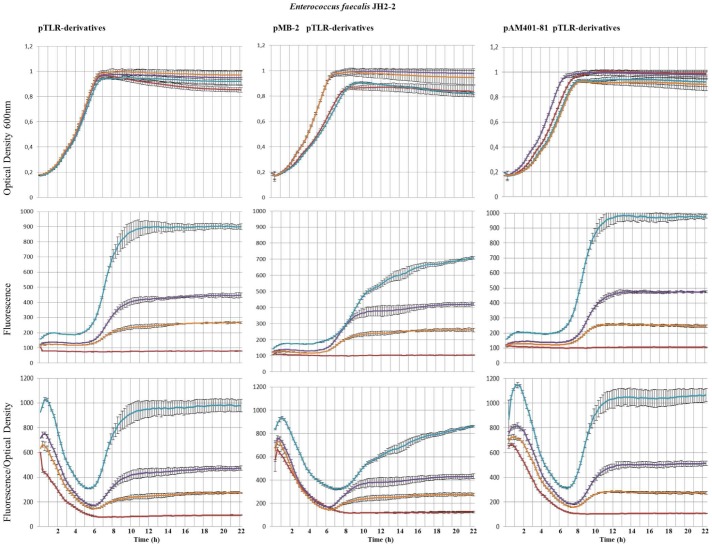
Influence of pMB-2 and pAM401-81 plasmids in the expression of the *as-48* cluster promoters during prolonged growth in CM-G medium normalized by the OD 600 nm in *E. faecalis* JH2-2 containing the pTLR1-derivatives (low panels). The growth of cultures was monitored at a wavelength of 600(upper panels). Fluorescence emission of mCherry was recorded at 620 nm after excitation at a wavelength of 590 nm (medium panels). pTLR1d (red), pLTR1-P_A_ (purple), pLTR1-P_2(2)_ (sky blue), pLTR1-P_D1_ (orange). Standard deviation bars for the different replicates are included.

It is likely that the promoters here identified could be regulated in the native *E. faecalis* S-48 strain by the presence of genes harbored in its genome or in pMB-2 (the native plasmid found in this strain) or even to be influenced by the presence of pAM401-81 (with only the *as-48* gene cluster cloned). Furthermore, an adapted response to the cultural conditions cannot be discarded in whichever condition. To address these questions, we have designed different experiments in *E. faecalis* to compare the fluorescence emitted during the growth of the JH2-2 transformants containing P_A_, P_2(2)_ or P_D1_ cloned into pTLR1, with that of the double transformants containing, additionally, either pAM401-81 or pMB-2, both of them compatible with pTLR1. In the results exposed in Figures 3 and 4, it could be observed that the presence of pAM401-81 or pMB-2 affects the expression of P_2(2)_ and P_A_ promoters (Figures 3 and 4). Thus, in presence of pAM401-81, P_2(2)_ reaches values of 1065 AU at 22 h, which are higher (*p* = 0.018) than those obtained for the single JH2-2(pLTR1-P_2(2)_) transformants, although the most noticeable result is the remarkably reduced fluorescence (*p* values between 0.004 and 0.000) repeatedly observed in presence of pMB-2. These results are also in accordance with the minor amounts of secreted AS-48 observed by JH2-2(pMB-2) compared to that of JH2-2(pAM401-81) transformants (Figure S1). In relation to P_A_ we found that the levels of fluorescence emitted by *E. faecalis* JH2-2 (pTLR1-P_A_) are slightly higher in presence of pAM401-81 but more reduced when pMB-2 is present, with significant differences after 14 h of growth (*p* value of 0.014) according to the statistical analysis performed.

**Figure 5:**
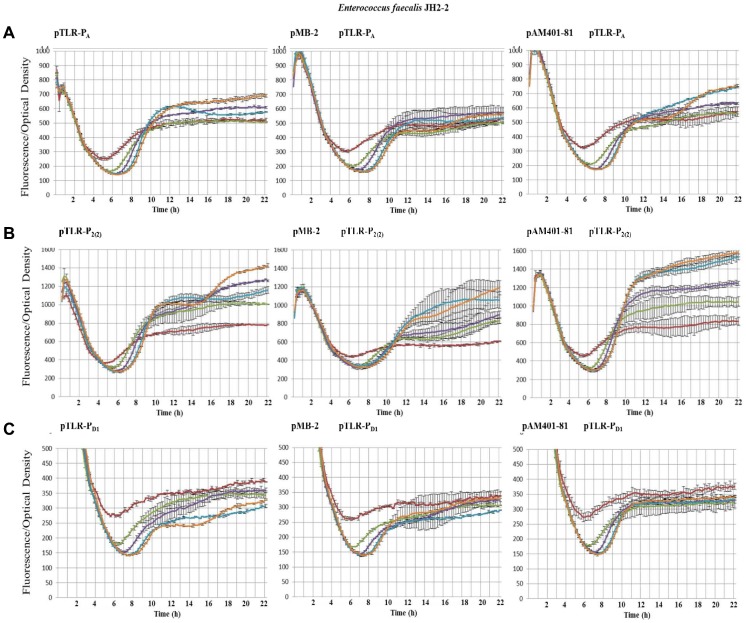
Expression of fluorescence of the mCherry normalized by the OD600 during prolonged growth in CM-G medium of *E. faecalis* JH2-2 containing the pTLR1-derivatives as indicated or double transformants containing pTLR1-derivatives and pMB-2 or pAM401-81 plasmids. Fluorescence emission of mCherry was recorded at 620(red), pH 6.5 (green), pH 7.0 (purple), pH 7.5 (sky blue), and pH 8.0 (orange) at a wavelength of 600 nm. Standard deviation bars for the different replicates are included.

Otherwise, the pH is an outstanding factor in LAB bacteria because of the production of lactic acid during the fermentative metabolism leads to the acidification of the media and the arrest of cell growth and, consequently, to the beginning of stationary phase. The influence of the pH in the production of different bacteriocins, including the circular sactipeptide subtilosin A, has been reported [15], . Consequently, we have investigated the influence of the pH on the levels of mCherry expression relative to the cell mass, in the single and double transformants during prolonged cultivation in CM-G broth buffered at pH values of 6.0, 6.5, 7.0, 7.5 and 8.0. The growth curves showed a similar profile in all the conditions tested. The highest OD values were achieved at the highest starting pH of the culture, reaching the stationary phase between 6 h and 10 h after inoculation (Figures S2, S3, S4). In overall, the mCherry expression driven from P_A_ and P_2(2)_ promoters confirmed that the highest expression levels are achieved at the high pHs (ca. 8) with significant results (*p* values of 0.000) from 16 and 18 h of growth (Figure 5 A and B), respectively, while P_D1_ seems to perform better at low pH (Figure 5C). Therefore, it is worth to emphasize that due to the presence of glucose in the medium the initial pH is only maintained during the first 6 h of growth and then, when the exponential growth commences, declined between 1.5−2 units in each case. These results justify the growth curves obtained and are in accordance with the importance of the pH stabilization at 6.5 during the growth as a key factor influencing the AS-48 production [41].

As above, the response of the *as-48* promoters showed an improved expression of the mCherry protein in presence of pAM401-81, particularly at the most alkaline pH (Figure 5). The most outstanding result was once more detected with P_2(2)_ in the presence of pMB-2 (Figure 5B). Promoter P_2(2)_ controls the expression of the *as-48C_1_DD_1_EFGH* genes encoding two ABC transporters for secretion (*as-48C_1_D*) and self-protection (*as-48EFGH*), in addition to the immunity determinant (*as-48D_1_*) against AS-48. As it can be seen in Figure 5B, expression of mCherry from P_2(2)_ was retarded in *E. faecalis* JH2-2 (pMB-2) and the fluorescence levels were visibly lower at any pH assayed. These results, that have been several times repeated, confirm the above suggestion on the P_2(2)_ promoter, in the sense that it could be regulated in a negative fashion by genes existing in the native pMB-2 plasmid different from those of the *as-48* cluster.

## Conclusions

The current study analyses the functionality of seven promoter regions (namely P_A_, P_C_, P_D1_ and two regions for P_2_ and for P_3_) putatively involved in the full expression of the AS-48 character, which is dependent on the co-ordinated expression of the *as-48ABCC_1_DD_1_EFGH* genes. Identifying promoters in this locus is relevant to understand how AS-48 is produced, and how to engineer strains to more effectively produce AS-48. The corresponding amplified regions were cloned into the promoter-probe vector pTLR1 by transcriptional fusions with the *mCherry* gene. The fluorescence emitted by the transformants with the pTLR1-derivatives during a prolonged incubation in CM-G medium, allowed us to ratify the existence of the P_A_ promoter (driving the expression of the *as-48ABC* operon) and, more importantly, to definitively localize the P_2(2)_ promoter (involved in the transcription of the second operon *as-48CC_1_DD_1_EFGH*), and the internal P_D1_ promoter, presumably responsible for the transcription of the last four genes (*as-48EFGH*) together with the immunity determinant *as-48D_1_*. The other promoter regions studied, included the P_3_ promoter reiteratively proposed [4−6], , could be discarded for the absence of functionality in the current assay.

Remarkably, the strongest promoter of the *as-48* cluster in LAB strains was the P_2(2)_ promoter here identified, which seems negatively regulated by genes present in pMB-2 plasmid and up-regulated by high pHs and genes of the *as-48* cluster, although having a strong basal activity in the absence of regulators. It is tempting to speculate with a membrane stress caused by AS-48 as the trigger for an increased transcription from P_2(2)_ that could involve alternative extracellular function σ factors. This stress could be controlled in cells containing pMB-2 by additional mechanisms encoded in this plasmid whereas in the cells transformed with pAM401-81 this function is overtaken by the two ABC-transporters coded in the *as-48C_1_DD_1_EFGH* operon. Additionally, the pMB-2 plasmid is a pheromone-responding plasmid involved in conjugation process between enterococcal communities in natural environments. Cells harboring pMB-2 have the capability to elicit a complex response to sex pheromones secreted by the receptor cells, inducing coordinated responses among members of a community and resulting in the formation of cell aggregates that allow clumping of cells to facilitate efficient conjugal transfer of this plasmid. Expression of genes involved in secretion of the bactericidal AS-48 bacteriocin, rapidly kill conventional recipient enterococcal cells preventing the conjugation process, being a disadvantage relative to the transfer of the pMB-2-plasmid from bacteriocin-producing donors, as it has been already demonstrated in a recent study about the transferability of R-Plasmid in bacteriocin-producing *E. coli* donors [44]. Interestingly, P_A_ and P_D1_ promoters have a strength that is one half and one quarter, respectively, compared to P_2(2)_. Both promoters drive a constitutive transcription in all the assay conditions, although the presence of the *as-48* genes and the pH seem to enhance their expression.

We also conclude that the strength of the *as-48* promoters is organism dependent. Thus, the strength of all these promoters was highest in *E. coli*, while in LAB strains only minor differences could be observed. Surprisingly the three promoters of the *as-48* cluster perform slightly better in lactococcal cells (Figure 2), indicating that this is not the reason that can justify the inability for the heterologous expression of AS-48 described by Fernández *et al.* [19] and supporting the idea of an incorrect processing of the mRNA or an inefficient production of the modification machinery involved in AS-48 maturation.

## Supporting Information

Figure S1 Antibacterial activity of JH2-2(pAM401-81) and JH2-2(pMB-2) against JH2-2 used as indicator strain.
(TIF)

Figure S2 Influence of pMB-2 and pAM401-81 plasmids in the expression of P_A_ promoter during prolonged growth in CM-G medium at different pH values normalized by the OD 600 nm in *E. faecalis* JH2-2 (pTLR1-P_A_) (low panels). The growth of cultures was monitored at a wavelength of 600 nm (upper panels). Fluorescence emission of mCherry was recorded at 620 nm after excitation at a wavelength of 590 nm (medium panels). pH 6 (red), pH 6.5 (green), pH 7.0 (purple), pH 7.5 (sky blue), and pH 8.0 (orange). Standard deviation bars for the different replicates are included.
(TIF)

Figure S3 Influence of pMB-2 and pAM401-81 plasmids in the expression of P_2(2)_ promoter during prolonged growth in CM-G medium at different pH values normalized by the OD 600 nm in *E. faecalis* JH2-2 (pTLR1-P_2(2)_) (low panels). The growth of cultures was monitored at a wavelength of 600 nm (upper panels). Fluorescence emission of mCherry was recorded at 620 nm after excitation at a wavelength of 590 nm (medium panels). pH 6 (red), pH 6.5 (green), pH 7.0 (purple), pH 7.5 (sky blue), and pH 8.0 (orange). Standard deviation bars for the different replicates are included.
(TIF)

Figure S4 Influence of pMB-2 and pAM401-81 plasmids in the expression of P_D1_ promoter during prolonged growth in CM-G medium at different pH values normalized by the OD 600 nm in *E. faecalis* JH2-2 (pTLR1-P_D1_) (low panels). The growth of cultures was monitored at a wavelength of 600 nm (upper panels). Fluorescence emission of mCherry was recorded at 620 nm after excitation at a wavelength of 590 nm (medium panels). pH 6 (red), pH 6.5 (green), pH 7.0 (purple), pH 7.5 (sky blue), and pH 8.0 (orange). Standard deviation bars for the different replicates are included.
(TIF)

Table S1 Sequence of promoter regions studied in this work. The predicted -10 and -35 sequences are underlined and depicted in bold.
(DOCX)

## References

[1] Khan H, Flint S, Pak-Lam Yu (2010) Enterocins in food preservation. Intern J Food Microbiol 141: 1–10. 10.1016/j.ijfoodmicro.2010.03.005 20399522

[2] Maqueda Abreu M, Martínez Bueno M, Valdivia Martínez E, Ananou Jaled S, Cebrián Castillo R (2012) Spanish patent P201231060-IPR-416- ES-2387425-B2.

[3] Maqueda M, Gálvez A, Martínez Bueno M, Sanchez-Barrena MJ, González C, et al (2004) Peptide AS-48: prototype of a new class of cyclic bacteriocins. Curr Prot Pept Sci 5: 399–416. 10.2174/1389203043379567 15544535

[4] Martínez-Bueno M, Gálvez A, Valdivia E, Maqueda M (1990) A transferable plasmid associated with AS-48 production in *Enterococcus faecalis* . J Bacteriol 172: 2817–2818. 2110152 10.1128/jb.172.5.2817-2818.1990 PMC208938

[5] Díaz M, Valdivia E, Martínez-Bueno M, Fernández M, Soler-González AS, et al (2003) Characterization of a new operon, *as-48EFGH*, from the *as-48* gene cluster involved in immunity to enterocin AS-48. Appl Environ Microbiol 69: 1229–1236. 12571051 10.1128/AEM.69.2.1229-1236.2003 PMC143590

[6] Tomita H, Fujimoto S, Tanimoto K, Ike Y (1997) Cloning and genetic and sequence analyses of the bacteriocin 21 determinant encoded on the *Enterococcus faecalis* pheromone-responsive conjugative plasmid pPD1. J Bacteriol 179: 7843–7855. 9401046 10.1128/jb.179.24.7843-7855.1997 PMC179750

[7] Abriouel H, Lucas R, Ben Omar N, Valdivia E, Maqueda M, et al (2005) Enterocin AS-48RJ: a variant of enterocin AS-48 chromosomally encoded by the food isolate *Enterococcus faecium* RJ16. Syst Appl Microbiol 28: 383–397. 16094865 10.1016/j.syapm.2005.01.007

[8] Cebrián R, Baños A, Valdivia E, Pérez-Pulido R, Martínez-Bueno M, et al (2012) Characterization of functional, safety, and probiotic properties of *Enterococcus faecalis* UGRA10, a new AS-48-producer strain. Food Microbiol 30: 59–67. 22265284 10.1016/j.fm.2011.12.002

[9] Martínez-Bueno M, Valdivia E, Galvez A, Coyette J, Maqueda M (1998) Analysis of the gene cluster involved in production and immunity the peptide antibiotic AS-48 in *Enterococcus faecalis* . Mol Microbiol 27: 347–358. 9484890 10.1046/j.1365-2958.1998.00682.x

[10] Fernández M, Sánchez-Hidalgo M, García-Quintáns N, Martínez-Bueno M, Valdivia E, et al (2008) Processing of the *as-48ABC* RNA in AS-48 enterocin production by *Enterococcus faecalis* . J Bacteriol 190: 240–250. 17981958 10.1128/JB.01528-07 PMC2223719

[11] Ruiz-Cruz S, Solano-Collado V, Espinosa M, Bravo A (2010) Novel plasmid-based genetic tools for the study of promoters and terminators in *Streptococcus pneumoniae* and *Enterococcus faecalis* . J Microbiol Methods 83: 156–163. 20801171 10.1016/j.mimet.2010.08.004

[12] Maqueda M, Sánchez-Hidalgo M, Fernández M, Montalbán-López M, Valdivia E, et al (2008) Genetic features of circular bacteriocins produced by Gram-positive bacteria. FEMS Microbiol Rev 32: 2–22. 18034824 10.1111/j.1574-6976.2007.00087.x

[13] Strauch MA, Bobay BG, Cavanagh J, Yao F, Wilson A, et al (2007) Abh and AbrB control of *Bacillus subtilis* antimicrobial gene expression. J Bacteriol 189: 7720–7732. 17720793 10.1128/JB.01081-07 PMC2168746

[14] Nakano MM, Zheng G, Zuber P (2000) Dual control of *sbo-alb* operon expression by the Spo0 and ResDE systems of signal transduction under anaerobic conditions in *Bacillus subtilis* . J Bacteriol 182: 3274–3277. 10809710 10.1128/jb.182.11.3274-3277.2000 PMC94517

[15] Leães FL, Velho RV, Caldas DG, Pinto JV, Tsai SM, et al (2013) Influence of pH and temperature on the expression of *sboA* and *ituD* genes in *Bacillus* sp. P11. Antonie Van Leeuwenhoek 104: 149–154. 23677688 10.1007/s10482-013-9935-z

[16] Cebrián R, Maqueda M, Neira JL, Valdivia E, Martínez-Bueno M, et al (2010) Insights into the functionality of the putative residues involved in enterocin AS-48 maturation. Appl Environ Microbiol 76: 7268–7276. 20833793 10.1128/AEM.01154-10 PMC2976261

[17] Montalbán-López M, Spolaore B, Pinato O, Martínez-Bueno M, Valdivia E, et al (2008) Characterization of linear forms of the circular enterocin AS-48 obtained by limited proteolysis. FEBS Letters 582: 3237–3242. 18760277 10.1016/j.febslet.2008.08.018

[18] Sánchez-Hidalgo M, Montalbán-López M, Cebrián R, Valdivia E, Martínez-Bueno M, et al (2011) AS-48 bacteriocin: close to perfection. Cell Mol Life Sci 68: 2845–2857. 21590312 10.1007/s00018-011-0724-4 PMC11115006

[19] Fernández M, Martínez-Bueno M, Martín MC, Valdivia E, Maqueda M (2007) Heterologous expression of enterocin AS-48 in several strains of lactic acid bacteria. J Appl Microbiol 102: 1350–1361. 17448170 10.1111/j.1365-2672.2006.03194.x

[20] García-Cayuela T, Mohedano ML, Pérez-Gómez de Cadiñanos L, Fernández de Palencia P, Boden D, et al.. (2011) Transcriptional-fusion vectors for detection of uni- and bidirectional promoter regions in lactic acid bacteria. Spanish patent P201130356.

[21] de Ruyter PG, Kuipers OP, de Vos WM (1996) Controlled gene expression systems for *Lactococcus lactis* with the food-grade inducer nisin. Appl Environ Microbiol 62: 3662–3667. 8837421 10.1128/aem.62.10.3662-3667.1996 PMC168174

[22] Mierau I, Kleerebezem M (2005) 10 years of the nisin-controlled gene expression system (NICE) in *Lactococcus lactis* . Appl Microbiol Biotechnol 68: 705–717. 16088349 10.1007/s00253-005-0107-6

[23] Mu D, Montalbán-López M, Masuda Y, Kuipers OP (2013) Zirex: a novel zinc-regulated expression system for *Lactococcus lactis* . Appl Environ Microbiol 79: 4503–4508. 23666339 10.1128/AEM.00866-13 PMC3697519

[24] García-Cayuela T, de Cadiñanos LP, Mohedano ML, de Palencia PF, Boden D, et al (2012) Fluorescent protein vectors for promoter analysis in lactic acid bacteria and *Escherichia coli* . Appl Microbiol Biotechnol 96: 171–181. 22534822 10.1007/s00253-012-4087-z

[25] Goel A, Santos F, de Vos WM, Teusink B, Molenaar D (2012) Standardized assay medium to measure *Lactococcus lactis* enzyme activities while mimicking intracellular conditions. Appl Environ Microbiol 78: 134–143. 22020503 10.1128/AEM.05276-11 PMC3255609

[26] Poolman B, Konings WN (1988) Relation of growth of *Streptococcus lactis* and *Streptococcus cremoris* to amino acid transport. J Bacteriol 170: 700–707. 3123462 10.1128/jb.170.2.700-707.1988 PMC210711

[27] Gálvez A, Maqueda M, Valdivia E, Quesada A, Montoya E (1986) Characterization and partial purification of a broad spectrum antibiotic AS–48 produced by *Streptococcus faecalis* . Can J Microbiol 32: 765–771. 3098396 10.1139/m86-141

[28] Seidman CE, Struhl K, Sheen J, Jessen T (1997) Transformation using calcium chloride. *In* Current protocols in molecular biology, supplement 37, 1.8.1. John Wiley & Sons, Inc.

[29] Holo H, Nes I (1995) Transformation of *Lactococcus* by electroporation. Methods Mol Biol 47: 195–199. 7550735 10.1385/0-89603-310-4:195

[30] Friesenegger A, Fiedler S, Devriese LA, Wirth R (1991) Genetic transformation of various species of *Enterococcus* by electroporation. FEMS Microbiol Lett 63: 323–327. 1905659 10.1016/0378-1097(91)90106-k

[31] Reese MG (2001) Application of a time-delay neural network to promoter annotation in the *Drosophila melanogaster* genome. Comput Chem 2: 51–56. 10.1016/s0097-8485(01)00099-7 11765852

[32] Solovyev V, Salamov A (2011) Automatic Annotation of Microbial Genomes and Metagenomic Sequences. In *Metagenomics and its Applications in Agriculture, Biomedicine and Environmental Studies* (Ed. R.W. Li), Nova Science Publishers, p. 61–78.

[33] Gomori G (1955) Preparation of buffers for use in enzyme studies. Meth Enzymol 1: 143–146.

[34] Voskuil MI, Chambliss GH (1998) The −16 region of *Bacillus subtilis* and other Gram-positive bacterial promoters. Nucleic Acids Res 26: 3584–3590. 9671823 10.1093/nar/26.15.3584 PMC147726

[35] Perez-Martin J, Rojo F, de Lorenzo V (1994) Promoters responsive to DNA bending: a common theme in prokaryotic gene expression. Microbiol Rev 58: 268–290. 8078436 10.1128/mr.58.2.268-290.1994 PMC372964

[36] Meng W, Belyaeva T, Savery NJ, Busby SJ, Ross WE, et al (2001) UP element dependent transcription at the *Escherichia coli rrnB* P1 promoter: positional requirements and role of the RNA polymerase alpha subunit linker. Nucleic Acids Res 29: 4166–4178. 11600705 10.1093/nar/29.20.4166 PMC60210

[37] Mu F, Masuda Y, Zendo T, Ono H, Kitagawa H, I, et al. (2013) Biological function of a DUF95 superfamily protein involved in the biosynthesis of a circular bacteriocin, leucocyclicin Q. J Biosci Bioeng doi: 10.1016/j.jbiosc.2013.06.023. 10.1016/j.jbiosc.2013.06.023 23906710

[38] Zhang J, Zhang Y, Liu SN, Han Y, Zhou ZJ (2012) Modelling growth and bacteriocin production by *Pediococcus acidilactici* PA003 as a function of temperature and pH value. Appl Biochem Biotechnol 166: 1388–1400. 22246730 10.1007/s12010-011-9532-4

[39] Guerra NP, Pastrana L (2003) Influence of pH drop on both nisin and pediocin production by *Lactococcus lactis* and *Pediococcus acidilactici* . Lett Appl Microbiol 37: 51–55. 12803556 10.1046/j.1472-765x.2003.01346.x

[40] Messens W, Neysens P, Vansieleghem W, Vanderhoeven J, De Vuyst L (2002) Modeling growth and bacteriocin production by *Lactobacillus amylovorus* DCE 471 in response to temperature and pH values used for sourdough fermentations. Appl Environ Microbiol 3: 1431–1435. 10.1128/AEM.68.3.1431-1435.2002 PMC123765 11872497

[41] Ananou S, Muñoz A, Gálvez A, Martínez-Bueno M, Maqueda M, et al (2008) Optimization of enterocin AS-48 production on a whey-based substrate. Int Dairy J 18: 923–927.

[42] Yagi Y, Clewell DB (1980) Recombination-deficient mutant of *Streptococcus faecalis* . J Bacteriol 143: 966–970. 6782083 10.1128/jb.143.2.966-970.1980 PMC294401

[43] Bourgeois PLF, Mata M, Ritzenthaler R (1991) Pulsed field gel electrophoresis as a tool studying the phylogeny and genetic history of lactococcal strains, p. 301. *In* Duny GM, Cleraly PP, McKay LL (ed) Genetic and Molecular Biology of Streptococci, Lactococci and Enterococci. American Society for Microbiology. Washington DC.

[44] Usui M, Hiki M, Murakami K, Ozawa M, Nagai H, et al (2012) Evaluation of transferability of R-Plasmid in bacteriocin-producing donors to bacteriocin-resistant recipients. Japan J Infect Dis 65: 252–255. 22627309 10.7883/yoken.65.252

