# Supplementary figures and images for: Analysis of the Promoters Involved in Enterocin AS-48 Expression
Source: PLoS One. 2014 Mar 4;9(3):e90603. doi: 10.1371/journal.pone.0090603 (PMC3942455; doi:10.1371/journal.pone.0090603)

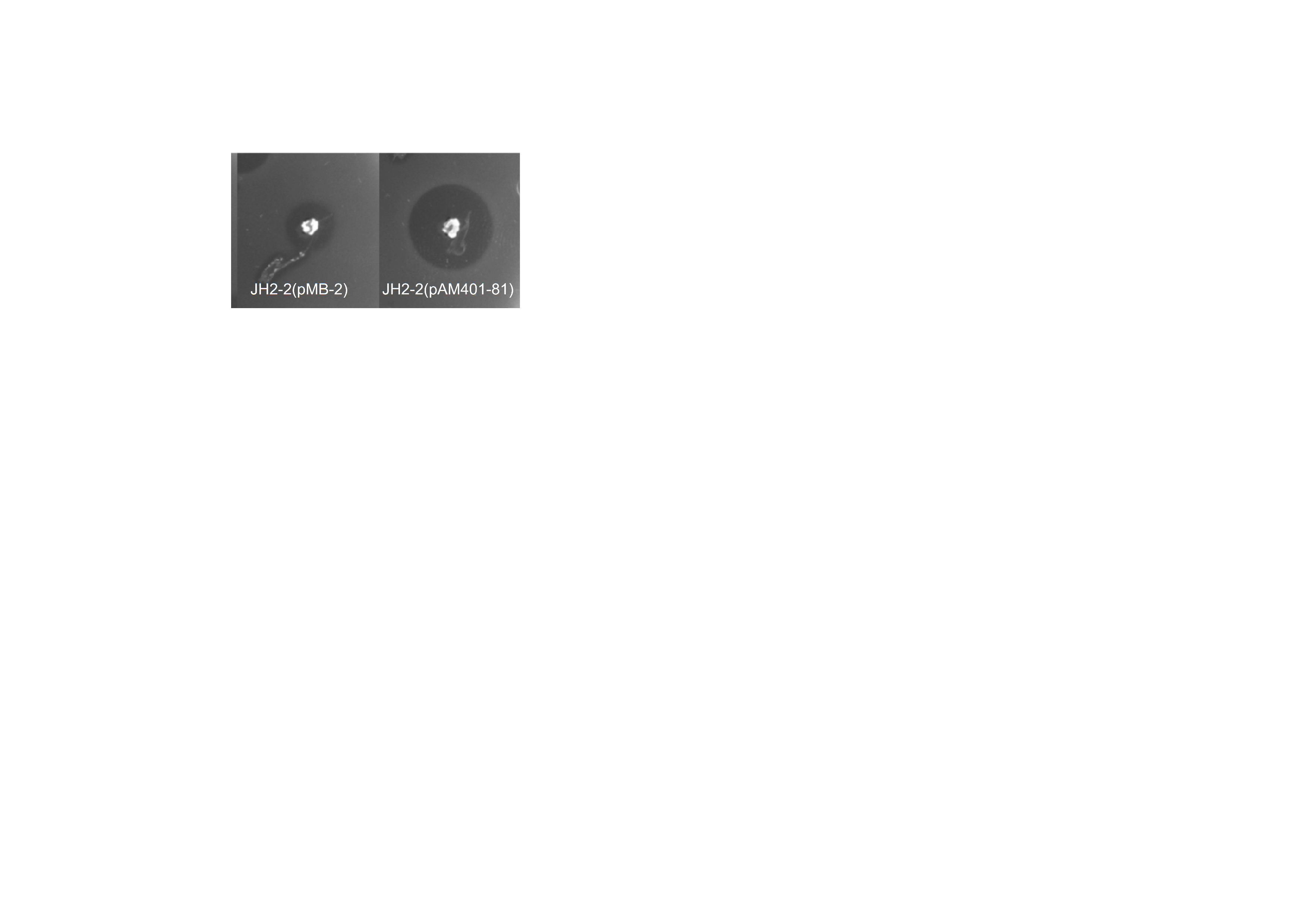

Supplement: Figure S1 — Antibacterial activity of JH2-2(pAM401-81) and JH2-2(pMB-2) against JH2-2 used as indicator strain. (TIF) [file pone.0090603.s001.tif]

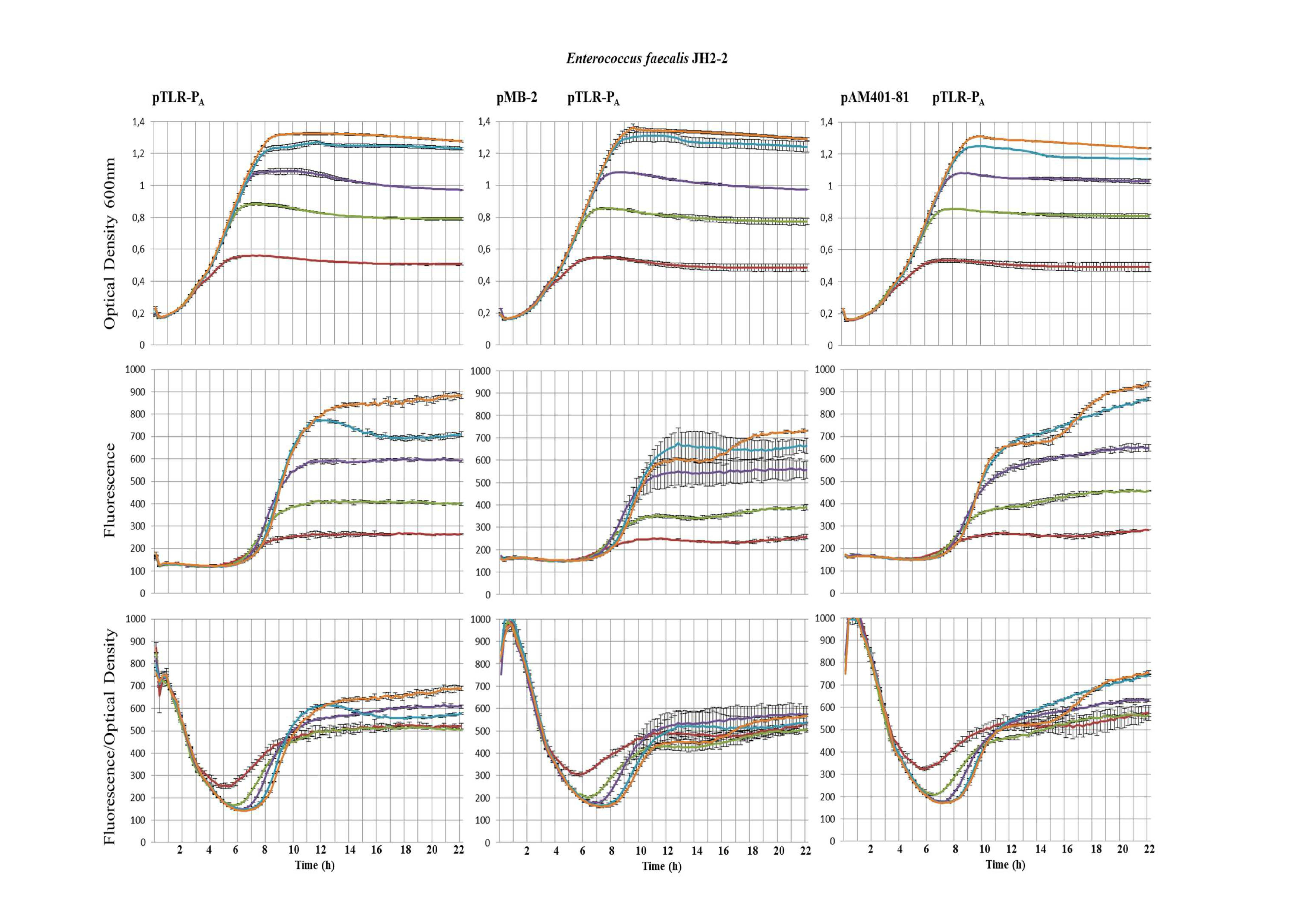

Supplement: Figure S2 — Influence of pMB-2 and pAM401-81 plasmids in the expression of PA promoter during prolonged growth in CM-G medium at different pH values normalized by the OD 600 nm in E. faecalis JH2-2 (pTLR1-PA) (low panels). The growth of cultures was monitored at a wavelength of 600 nm (upper panels). Fluorescence emission of mCherry was recorded at 620 nm after excitation at a wavelength of 590 nm (medium panels). pH 6 (red), pH 6.5 (green), pH 7.0 (purple), pH 7.5 (sky blue), and pH 8.0 (orange). Standard deviation bars for the different replicates are included. (TIF) [file pone.0090603.s002.tif]

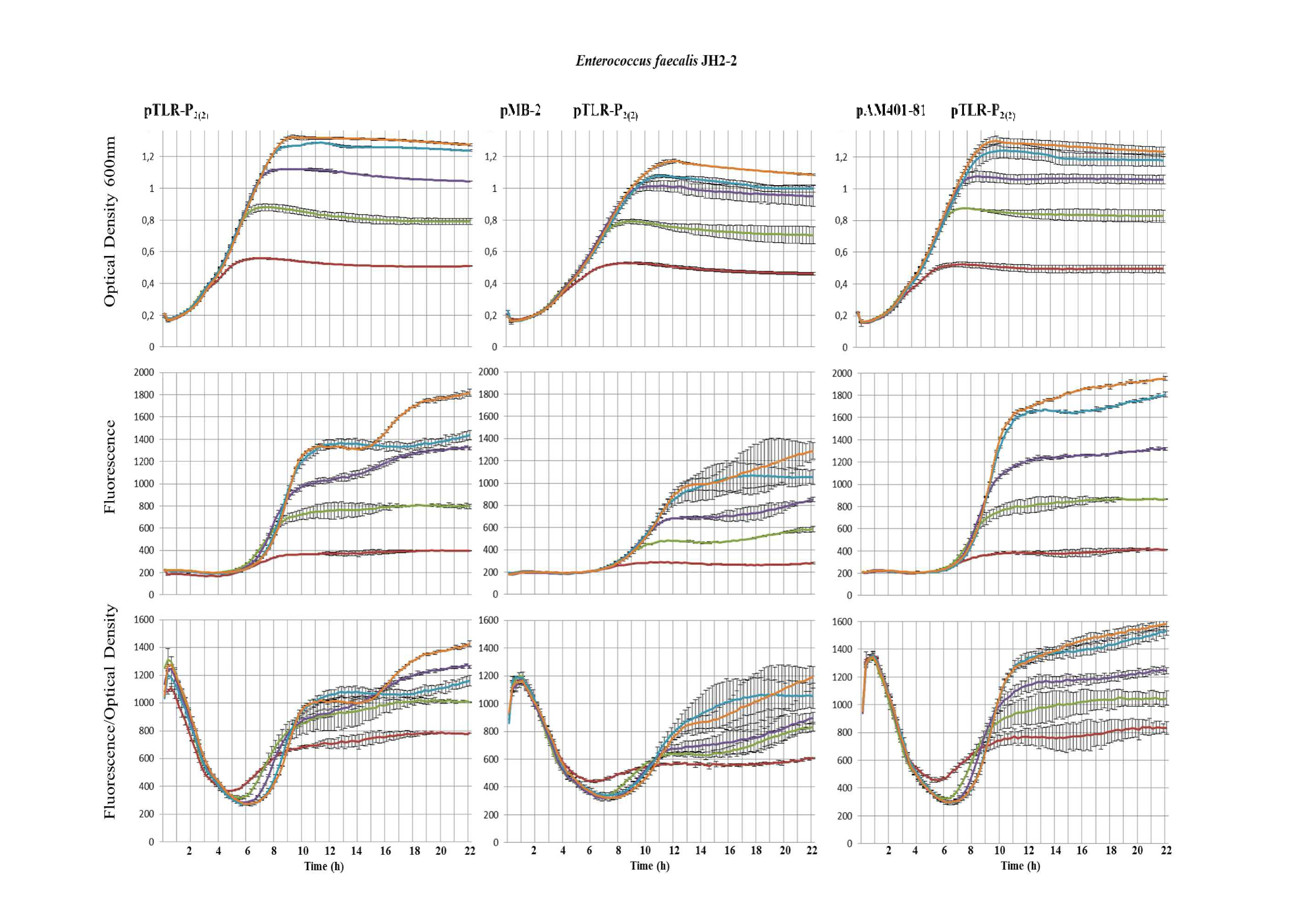

Supplement: Figure S3 — Influence of pMB-2 and pAM401-81 plasmids in the expression of P2(2) promoter during prolonged growth in CM-G medium at different pH values normalized by the OD 600 nm in E. faecalis JH2-2 (pTLR1-P2(2)) (low panels). The growth of cultures was monitored at a wavelength of 600 nm (upper panels). Fluorescence emission of mCherry was recorded at 620 nm after excitation at a wavelength of 590 nm (medium panels). pH 6 (red), pH 6.5 (green), pH 7.0 (purple), pH 7.5 (sky blue), and pH 8.0 (orange). Standard deviation bars for the different replicates are included. (TIF) [file pone.0090603.s003.tif]

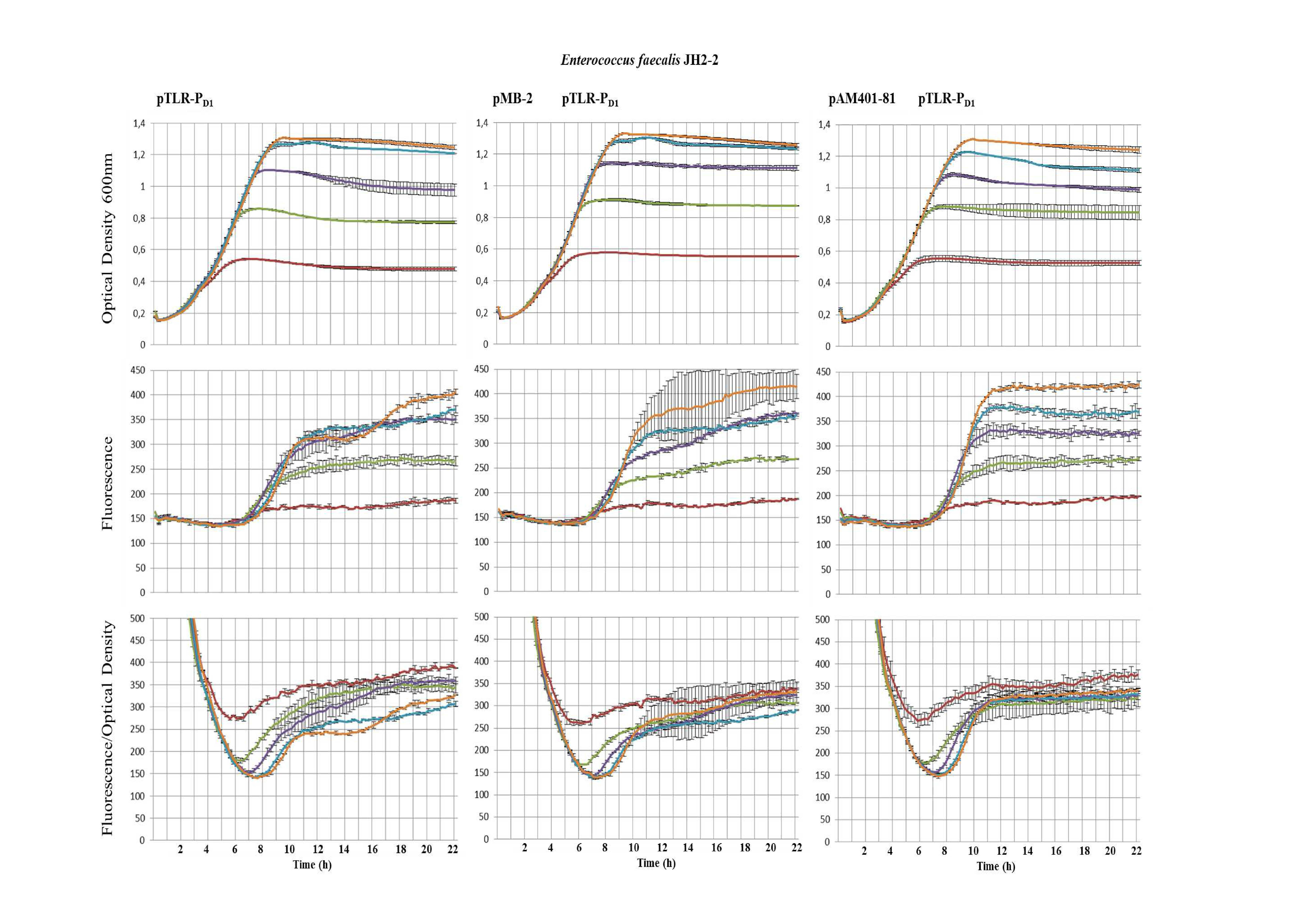

Supplement: Figure S4 — Influence of pMB-2 and pAM401-81 plasmids in the expression of PD1 promoter during prolonged growth in CM-G medium at different pH values normalized by the OD 600 nm in E. faecalis JH2-2 (pTLR1-PD1) (low panels). The growth of cultures was monitored at a wavelength of 600 nm (upper panels). Fluorescence emission of mCherry was recorded at 620 nm after excitation at a wavelength of 590 nm (medium panels). pH 6 (red), pH 6.5 (green), pH 7.0 (purple), pH 7.5 (sky blue), and pH 8.0 (orange). Standard deviation bars for the different replicates are included. (TIF) [file pone.0090603.s004.tif]
