# Supplementary material for: Analysis of the Promoters Involved in Enterocin AS-48 Expression
Source: PLoS One. 2014 Mar 4;9(3):e90603. doi: 10.1371/journal.pone.0090603 (PMC3942455; doi:10.1371/journal.pone.0090603)
Supplement: Table S1 — Sequence of promoter regions studied in this work. The predicted -10 and -35 sequences are underlined and depicted in bold. (DOCX) [file pone.0090603.s005.docx]

| **Promoter** | **Sequence** |
| --- | --- |
| P_A_ | GCCATGATTGATGAAAAAAATTCTGAAAATTATCTTTATGATATAGATATCGTAGAAGAAAACAAAGAAGTAGTTCAAGCGTATAACCAGGATAGAATGAAAGATAAGTCAAATGAAAATGAACAATTTGAAGGAATTAGTCATGATGTTGATTTCTAAGAAGTGTCTAGTTATTTTTTCTTGGGTTATTTACAGGAATAGATATGTTATTGC**TTGCAT**CAAAATAAACTACATGGG**TATAAT**AGCAATGAAATGCA |
| P_C_ | GTACATGCGATTAGATACCATTAATTTTGAGAAATATTTGAAAATTTCCTTCTATATATCAATATTTATTTT**TTTTCT**GGGAGTGTTAGTAGGAAT**TATAAT**AGGTCCTCATATAAATAAATTGGACTATTTTGGTCAGGAAGTTTCATTTTATAGTGTTAGTATTAATAATTTAAAGGTTTCTTTTTATTTCCTCACTATAGGAATGGTAACAGGGGGGATTTATGCATTTTTATTTATGGGTATAAATGGTTATATAATTGGTAAGTTGATTCAATATTTATACATTAATAATGAACTAAATATTTTGTATAAAGGTCTTCTTCCACATTTTTTTATAGAACTTTTAG |
| P_2(1)_ | TGCTGAGTTAAAGGTATACTCATTAAAGGAAGAGTGAACACTATTTTAAATTTATACAACCAAAGTAAAATTTTCGTGAATTATCTTTATAATAATATGTCGGTTACTTAAAATTTTTGAAATTCACAATAAACATATAATTAACAGTGCAATAATTTATTTGTCTACAAGTCAA**TTGGGA**TAGGCAACTATATTCGCA**TAAAAT**ATCTCTCACAAAAAGTGTCTCATCTATTGAATCTGATCCATATAAGTTCTTAAGAAAGG |
| P_2(2)_ | TGCTGAGTTAAAGGTATACTCATTAAAGGAAGAGTGAACACTATTTTAAATTTATACAACCAAAGTAAAATTTTCGTGAATTATCTTTATAATAATATGTCGGTTACTTAAAATTTTTGAAATTCACAATAAACATATAATTAACAGTGCAATAATTTATTTGTCTACAAGTCAATTGGGATAGGCAACTATATTCGCATAAAATATCTCTCACAAAAAGTGTCTCATCTATTGAATCTGATCCATATAAGTTCTTAAGAAAGGAAGATAAGTTTGCATAAGTTTACTTTGAAAAAGTGCATCGGTATTAC**TTCACT**ATTTTTTTTGTTTTCAAATAT**TTTAAT**TGCTGAAGAGTTTATTTTTGTAGAAAAAAACTTGAGTTTTTTTCCTAAATTAG |
| P_D1_ | GAATATGACGGCACATTGTATACAGAAAAAGTAACTAAAGAAAAAGTAGCTTTTATACCCTATAAAACTAAGCTATATCCTTATCTTGATGTTTTTGATCATATAAAGCTAATAGCAGAATTATGGGGAATTAAAACAGACTATTTAGAGTATAAAAGAAAAGTATTAGAATATTGTAACCGTCTAAACTTGGACTACTATAATAAGAGAGTAGAGTCTTACTCTACAGGTATGGAGTATAAACTATACATTTCTTTAATGTTGGCAAGAGATGTTTCTCTTGTATTATTAGATGAACCTTTTACCATGTTAGATAAAAAAAGTCGCTATTTAGCTATGGACTTAATCAAAGAGAAAAAAATAATAACAATATTTTCTTCACATCAGAAAGATA**TTGTAG**AATATTTGTCAAATGA**TATAAT**TAATCTTGACAAACTGAA |
| P_3(1)_ | AAAATAAGAAGCTGTACAATAGAATTACACTACTTTTTGGTAATTTTTA**TAGACT**AATCAGCAAAGGGAGTATGTA**AATAAT**TTTATTTATGTAACAATATCTATTAGAGGAAACCATATGAATTATGAAATCTTAGGAAGCTTAGTTAAAATTTATTTTTTAGATAGAATTCTTTAATTATGACAAGAAAG |
| P_3(2)_ | GAATTGATTACATTATTATTATAGTCTCACTATTAGCAACAATAGTCGCAATATTTTTAATAGGGATAGATTCTATGTTAGGAAAGATTTTTTTAGCTATTTCTCTCGG**TTTTTT**TTCTTCCCCATTCT**TAAAAT**GGATAAATAAATTAATAAATAAAAATTAAAAAAATAAGAAGCTGTACAATAGAATTACACTACTTTTTGGTAATTTTTATAGACTAATCAGCAAAGGGAGTATGTAAATAATTTTATTTATGTAACAATATCTATTAGAGGAAACCATATGAATTATGAAATCTTAGGAAGCTTAGTTAAAATTTATTTTTTAGATAGAATTCTTTAATTATGACAAGAAAG |

Table S1. Sequence of promoter regions studied in this work. The predicted -10 and -35 sequences are underlined and depicted in bold.
